# Fish Intake and Ovarian Cancer Risk: A Meta-Analysis of 15 Case-Control and Cohort Studies

**DOI:** 10.1371/journal.pone.0094601

**Published:** 2014-04-14

**Authors:** Pei-yue Jiang, Zhong-bo Jiang, Ke-xin Shen, Ying Yue

**Affiliations:** 1 Department of Gynecological Oncology, First Hospital of Jilin University, Changchun, China; 2 Department of Gastroenterology, China-Japan Union Hospital of Jilin University, Changchun, China; 3 Department of Colorectal and Anal Surgery, China-Japan Union Hospital of Jilin University, Changchun, China; Duke Cancer Institute, United States of America

## Abstract

**Background:**

Previous epidemiological studies have shown that fish consumption may modify the risk of ovarian cancer. However, these studies yielded controversial results. The present meta-analysis was undertaken to evaluate the relationship between fish intake and ovarian cancer risk.

**Methods:**

A literature search was carried out using Pubmed, Embase, and Cochrane Library Central database for all relevant studies up to August 2013. We pooled the relative risks (RR) from individual studies using fixed-effect or random-effect model, and carried out heterogeneity and publication bias analyses.

**Results:**

A total of 15 (ten case–control, and five cohort) studies were included in the present meta-analysis, representing data for 889,033 female subjects and 6,087 ovarian cancer cases. We found that total fish intake was not significantly associated with the risk of ovarian cancer among cohort studies (RR = 1.04 95% CI [0.89, 1.22]) as well as case–control studies (RR = 0.90, 95% CI [0.73,1.12]). There was no evidence of publication bias as suggested by Begg's test (P = 0.55) and Egger's test(P = 0.29).

**Conclusions:**

The present meta-analysis showed that total fish consumption was not significantly associated with the risk of ovarian cancer. Further analysis on different fish species and food preparation methods should be conducted in future studies.

## Introduction

Ovarian cancer is the most lethal gynecological malignancy and ranks as the sixth common cancer in women worldwide [1]. Less than 50% of patients are alive 5 years after initial diagnosis because the majority of cases are diagnosed with ovarian cancer at an advanced stage[2], [3]. Parity, oral contraceptive use, and family history are well-known risk factors for ovarian cancer[2], [4], [5]. However, the relationship between diet and the risk of ovarian cancer is still controversial.

Previous studies suggested that there was a significant association between meat intake, in particular red meat and processed meat, and the risk of several types of cancers, such as colorectal adenomas, esophageal cancer, and bladder cancer[6]–[8]. Fish is an important aspect of diet that has been linked favorably or unfavorably to the risk of several cancers[9]–[11]. The polyunsaturated omega-3 fatty acids in fish are thought to be able to reduce the risk of some types of cancers[12], however, on the other hand, carcinogenic and mutagenic Nnitroso compounds and heterocyclic amines in processed fish may increase the risk of cancer[13], [14]. There are several case-control and cohort studies investigating the association between fish intake and ovarian cancer risk, however, their results were inconsistent. In 2010, Kolahdooz et al [15]performed a meta-analysis of observational studies which investigated the association between meat, fish intake and ovarian cancer risk. They reported that high fish intake was associated with a borderline significantly reduced risk of ovarian cancer(RR = 0.84; 95% CI: 0.68, 1.03). However, their meta-analysis only included 8 observational studies, including two cohort studies, three hospital-based case-control studies, and three population-based case-control studies. Further more, their meta-analysis was too simple, and they haven't done sub-group analyses according to geographic location and adjustment factors, sensitivity analysis, and meta-regression analysis. Hence, we now performed a more detailed meta-analysis of observational studies to evaluate the effect of fish consumption on the risk of developing ovarian cancer.

## Materials and Methods

### Data Sources and Searches

The present meta-analysis was conducted following the Preferred Reporting Items for Systematic reviews and Meta-Analyses guidelines(PRISMA)[16], and the meta-analysis of observational studies in epidemiology (MOOSE) guidelines[17]. A literature search was carried out using Pubmed, Embase, and Cochrane Library Central database for all relevant studies published in English-language journals up to August 2013. Search terms included: “fish” or “seafood” and “ovarian” or “ovary” and “cancer” or “tumour” or “neoplasm” or “malignancy”. The reference lists of each comparative study included in this meta-analysis and previous reviews were manually examined to identify additional relevant studies.

### Study selection criteria

Two reviewers independently selected eligible observational studies that investigated fish intake and ovarian cancer risk. Disagreement between the two reviewers was settled by discussing with the third reviewer. Inclusion criteria were: (i) used a case-control or cohort study design; (ii) evaluated the association between fish intake and ovarian cancer risk; (iii) presented odds ratio (OR), relative risk (RR), or hazard ratio (HR) estimates with its 95% confidence interval (CI). Exclusion criteria were (i) lack of available data (ii) reviews, editorials, comments, reports from scientific sessions or discussions. When there were multiple publications from the same population, only data from the most recent report was included in the meta-analysis and the remaining publications were excluded. Studies reporting different measures of RR like risk ratio, rate ratio, hazard ratio, and odds ratio were included in the meta-analysis. In practice, these measures of effect yield a similar estimate of RR, since the absolute risk of ovarian cancer is low.

### Data extraction and methodological quality assessment

The following data was collected by two reviewers independently using a purpose-designed form: name of the first author, publishing time, country of the population studied, study design, study period, number of cancer cases and subjects, dietary assessment method, type of fish, the study-specific adjusted ORs, RRs, or HRs with their 95% CIs for the highest category of fish consumption versus the lowest, confounding factors for matching or adjustments. We used Newcastle-Ottawa scale to assess the methodologic quality of cohort and case-control studies. The Newcastle-Ottawa Scale contains eight items that are categorized three categories: selection (four items, one star each), comparability (one item, up to two stars), and exposure/outcome (three items, one star each). A “star” presents a “high-quality” choice of individual study. The full score was 9 stars, and the high-quality study was defined as a study with ≥6 awarded stars.

### Statistical analysis

The study-specific adjusted RRs were used as the common measure of association across studies. Because the absolute risk of ovarian cancer is low in human, the ORs in case–control studies should approximate the RRs or HRs; therefore, we reported all results as RRs for simplicity. Heterogeneity was assessed using the Cochran Q and I^2^ statistics. For the Q statistic, a P value<0.10 was considered statistically significant for heterogeneity; for the I^2^ statistic, heterogeneity was interpreted as absent (I^2^: 0%–25%), low (I^2^: 25.1%–50%), moderate (I^2^: 50.1%–75%), or high (I^2^: 75.1%–100%)[18]. Some studies presented individual risk estimates according to the different types of fish and did not report the effect of total fish consumption. In this situation, the study-specific effect size in overall analysis was calculated by pooling the risk estimates of the various fish types, using the inverse-variance method. Subgroup analyses were carried out according to (i) study design (cohort studies versus case-control studies), (ii)geographic location (Europe versus North America versus Asia versus Australia), (iii) number of adjustment factors (n≥8 versus n≤7), adjustment for alcohol intake (yes, no), adjustment for total energy intake (yes, no), adjustment for use of oral contraceptives(yes, no), adjustment for parity(yes, no), adjustment for smoking status(yes, no), adjustment for family history of ovarian cancer(yes, no), Pooled RR estimates and corresponding 95% CIs were calculated using the inverse variance method. When substantial heterogeneity was detected(I^2^≥50%), the summary estimate based on the random-effect model (DerSimonian-Laird method)[19] was reported, which assumed that the studies included in the meta-analysis had varying effect sizes. Otherwise, the summary estimate based on the fixed-effect model (the inverse variance method)[20] was reported, which assumed that the studies included in the meta-analysis had the same effect size. We carried out sensitivity analyses by excluding one study at a time to explore whether the results were significantly influenced by a specific study. To better investigate the possible sources of between-study heterogeneity, a meta-regression analysis was performed[21]. A univariate model was established, and then variables with P values ≥0.1 were entered into a multivariable model. Publication bias was assessed using Begg and Mazumdar adjusted rank correlation test and the Egger regression asymmetry test[22], [23]. All analyses were performed using Stata version 11.0 (StataCorp, College Station, TX).

## Results

### Literature search and study characteristics

**Figure 1:**
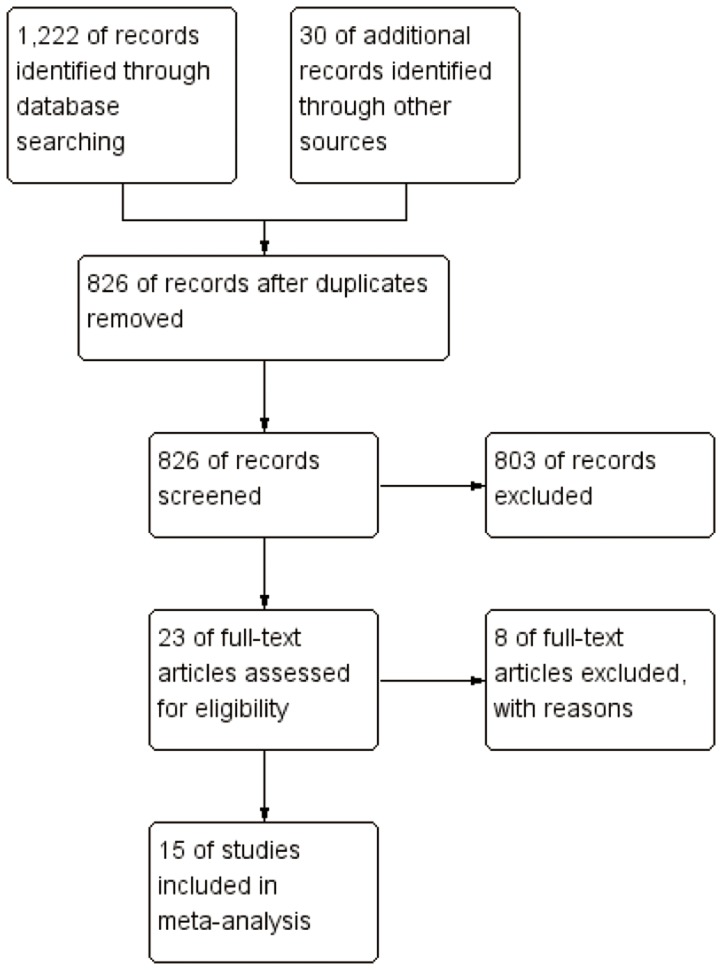
Flow diagram of screened, excluded, and analyzed publications.

Figure 1 illustrates the search process and the final selection of relevant studies. 1,222 records were identified through database searching, and 30 additional records were identified through other sources. On the basis of the titles and abstracts, we identified 23 full-text articles. After further evaluation, eight studies were excluded for lack of available data. At last, a total of 15 eligible studies published between 1984 and 2011 were identified, including 10 case–control studies[12], [15], [24]–[31] and five cohort studies[32]–[36] (Baseline data and other details of included case–control studies and cohort studies are shown in Table 1 and Table 2, respectively). Among the ten case-control studies, five studies were population based, and the other five studies were hospital based. A total of 889,033 female subjects, including 6,087 ovarian cancer cases were involved. Of the 15 included studies, six studies were conducted in Europe, three studies in Asia, five studies in North America, and remaining one in Australia. Most studies used food frequency questionnaires(FFQ) for the assessment of fish consumption. Most studies matched or adjusted for some potential confounders, including age, total energy intake, and use of oral contraceptives. Table S1 summarizes the quality scores of cohort studies and case-control studies. The Newcastle-Ottawa Scale scores for the included studies ranged from 4 to 9, with a median 7.5. The median scores of cohort studies and case-control studies were 9 and 5.5, respectively. 10 studies were deemed to be of a high quality (≥6).

**Table 1:** Characteristics of 10 case-control studies included in the meta-analysis.

Authors	Publication year	Study design	Study period	Country	Cases/Subjects	Type of fish	Variables of adjustment	Methods used for assessing fish intake	Control source
Kolahdooz F	2010	Case-control	1990–1993	Australia	793/2,984	Total fish, fatty fish, nonfatty fish	age, oral contraceptive use, level of education, parity, and energy intake	FFQ	population
Hu J	2008	Case-control	1994–1997	Canada	442/5,481	Total fish, fresh fish, smoked fish	age, province, education, BMI, alcohol use, pack-year smoking, total of vegetable and fruit intake, and total energy intake	FFQ	population
Pan SY	2004	Case-control	1994–1997	Canada	442/2,577	Total fish	age, province of residence, education, alcohol consumption, cigarette pack-years, BMI, total caloric intake, recreational physical activity, number of live births, menstruation years, and menopause status.	self-administered questionnaire	population
Yen ML	2003	Case-control	1993–1998	Taiwan	86/455	Total fish	age, income during marriage, and education	FFQ	hospital
Zhang M	2002	Case-control	1999–2000	China	254/906	Total fish	age, education, living area, BMI, smoking, alcohol drinking, tea drinking, family income marital and menopause status, parity, tubal ligation, oral contraceptive use, physical activity, family history of ovarian cancer, total energy intake, meat, vegetable, fruit, egg, milk intake	FFQ	hospital
Bosetti C	2001	Case-control	1992–1999	Italy	1,031/3,442	Total fish	age, study center, education, year of interview, parity, oral contraceptive use, meat, vegetable intake and energy intake	FFQ	population
Fernandez E	1999	Case-control	1983–1996	Italy	971/8,961	Total fish	age, area of residence, education, smoking, alcohol consumption, and BMI	FFQ	hospital
Mori M	1988	Case-control	1980–1986	Japan	110/330	Total fish	age	a uniform questionnaire	hospital
La Vecchia C	1987	Case-control	1983–1986	Italy	455/1,840	Total fish	age, interviewer, marital status, social class, education, parity, age at first birth, age at menarche, menopausal status, age at menopause, BMI, oral contraceptive and other female hormone use, retinol and carotene indices, added score of fat consumption and alcohol intake, meat, vegetable intake	FFQ	hospital
Cramer DW	1984	Case-control	1978–1981	USA	215/430	Total fish	age, race, residence	FFQ	population

BMI  =  body mass index; FFQ  =  food frequency questionnaire.

**Table 2:** Characteristics of five cohort studies included in the meta-analysis.

Authors	Publication year	Study design	Study period	Country	Cases/Subjects	Type of fish	Variables of adjustment	Methods used for assessing fish intake
Gilsing AM	2011	Cohort	1986–2002	Netherlands	55/62,573	Total fish	age, total energy intake, parity, and use of oral contraceptives	FFQ
Daniel CR	2011	Cohort	1995–1996	USA	758/198,720	Total fish	age, red meat intake, education, marital status, family history of cancer, race, BMI, smoking status, frequency of vigorous physical activity, menopausal hormone therapy in women, and intake of alcohol, fruit, vegetables, and total energy, poultry	FFQ
Schulz M	2007	Cohort	1992–2000	10 European countries: Denmark, France, Germany, Greece, Italy, the Netherlands, Norway, Spain, Sweden, United Kingdom	116/520,042	Total fish	BMI, parity, menopausal status, ever use of oral contraceptives, total energy intake, education, smoking, unilateral ovariectomy, and hormone replacement therapy use at baseline	FFQ
Kiani F	2006	Cohort	1976–1992	USA	71/13,281	Total fish	age, parity, BMI, age at menopause and hormone replacement therapy, and the stipulated dietary variables	a detailed lifestyle questionnaire including a dietary assessment
Larsson SC	2005	Cohort	1987–2004	Sweden	288/66,651	Total fish	age, BMI, educational level, parity, use of oral contraceptives and postmenopausal hormones, total energy intake, and quartiles of consumption of fruits, vegetables, and dairy products.	FFQ

BMI  =  body mass index; FFQ  =  food frequency questionnaire.

### Meta-Analysis results of case-control studies

**Figure 2:**
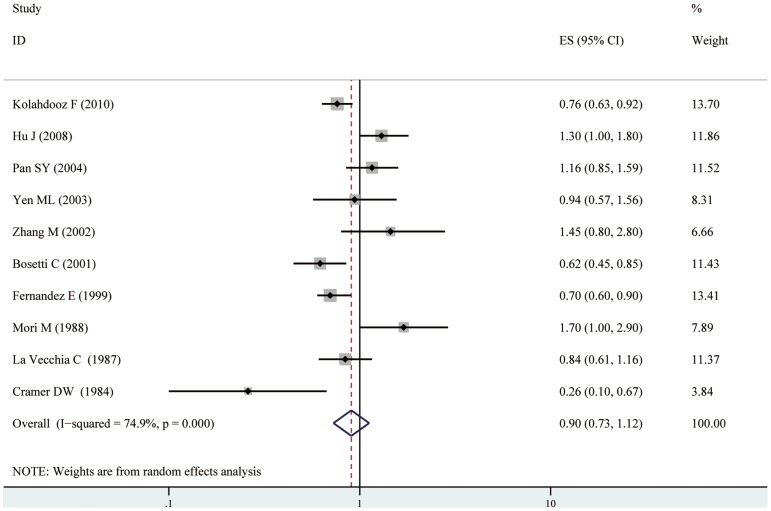
Forest plot: fish consumption and ovarian cancer risk among case-control studies. Squares indicated study-specific risk estimates (size of square reflects the study-statistical weight, i.e. inverse of variance); horizontal lines indicate 95% confidence intervals; diamond indicates summary relative risk estimate with its corresponding 95% confidence interval.

Because significant heterogeneity was observed (I^2^ = 74.9%, p<0.001), a random-effect model was chosen over a fixed-effect model and we found that fish consumption did not significantly affect the risk of ovarian cancer among case-control studies(RR = 0.90, 95% CI [0.73,1.12]). Both multivariable adjusted RR estimates with 95% CIs of each study and combined RR are shown in Figure 2. In a stratified analysis by control source, we haven't found significant association between fish consumption and ovarian cancer risk among population-based studies or hospital-based studies(RR = 0.82, 95% CI [0.58, 1.16], RR = 0.99, 95% CI [0.72, 1.36], respectively, presented in Table 3). When we stratified the various studies by geographic location, we found that fish consumption was associated with a significant reduced risk of ovarian cancer among studies conducted in Europe (RR = 0.71, 95%CI [0.61, 0.82]), and Australia (RR = 0.76, 95%CI [0.63, 0.92]). However, no significant association was detected among studies conducted in North America (RR = 0.91, 95%CI [0.53, 1.57]), and Asia (RR = 1.30, 95%CI [0.90, 1.88]). When we examined whether the associations were affected by adjustment for total energy intake, use of oral contraceptives, parity, smoking status, alcohol consumption, family history of ovarian cancer, the associations were significantly affected by use of oral contraceptives and parity. Further, it was observed that studies with higher control for potential confounders (n≥8) as well as studies with lower control (n≤7) presented no significant association between fish intake and ovarian cancer risk (shown in Table 3). To test the robustness of association and characterize possible sources of statistical heterogeneity, sensitivity analyses were carried out by excluding studies one-by-one and analyzing the homogeneity and effect size for all of rest studies. Sensitivity analysis indicated that no significant variation in combined RR by excluding any of the study, confirming the stability of present results.

**Table 3:** Summary risk estimates of the association between fish consumption and ovarian cancer risk among case-control studies.

	No. of studies	Pooled estimate		Tests of heterogeneity	
		RR	95% CI	P value	I^2^(%)
All studies	10	0.90	0.73–1.12	<0.001	74.9
Geographic location					
Europe	3	0.71	0.61–0.82	0.413	0.0
North America	3	0.91	0.53–1.57	0.007	80.1
Asia	3	1.30	0.90–1.88	0.261	25.5
Australia	1	0.76	0.63–0.92	N/A	N/A
Control source					
Population-based	5	0.82	0.58–1.16	<0.001	82.5
Hospital-based	5	0.99	0.72–1.36	0.012	69.1
Adjusted for confounders					
Number of adjustment factors					
n≥8 confounders	5	1.00	0.74–1.35	0.005	73.4
n≤7 confounders	5	0.81	0.60–1.10	0.005	73.3
Major confounders adjusted					
Total energy intake					
yes	5	0.97	0.72–1.31	0.001	79.1
no	5	0.83	0.58–1.19	0.004	73.6
Use of oral contraceptives					
yes	4	0.79	0.63–0.99	0.112	50.0
no	6	0.96	0.67–1.37	<0.001	81.0
Parity					
yes	4	0.79	0.63–0.99	0.112	50.0
no	6	0.96	0.67–1.37	<0.001	81.0
Alcohol consumption					
yes	5	0.79	0.55–1.13	0.003	74.9
no	5	1.01	0.76–1.35	0.002	76.1
Smoking status					
yes	4	1.07	0.74–1.55	0.001	84.7
no	6	0.80	0.60–1.06	0.006	69.0
Family history of ovarian cancer					
yes	1	1.45	0.78–2.27	N/A	N/A
no	9	0.87	0.70–1.09	<0.001	75.8

CI, confidence interval; N/A, not available; RR, relative risk.

### Meta-Analysis results of cohort studies

**Figure 3:**
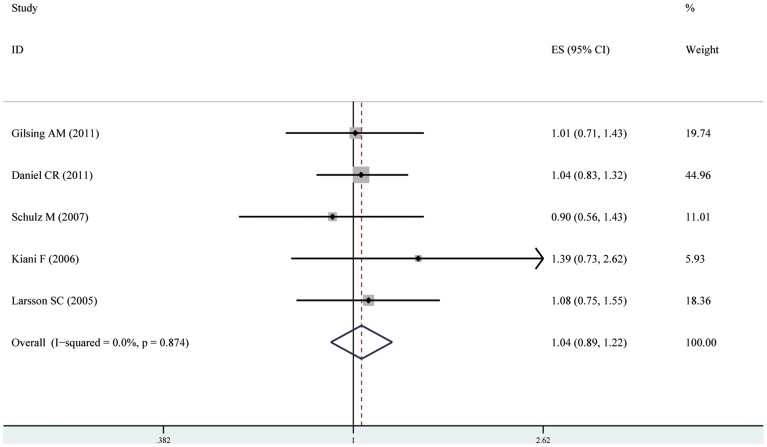
Forest plot: fish consumption and ovarian cancer risk among cohort studies. Squares indicated study-specific risk estimates (size of square reflects the study-statistical weight, i.e. inverse of variance); horizontal lines indicate 95% confidence intervals; diamond indicates summary relative risk estimate with its corresponding 95% confidence interval.

Because no significant heterogeneity was observed (I^2^ = 0%, p = 0.874), a fixed-effects model was chosen over a random-effects model and we found that fish consumption did not significantly affect the risk of ovarian cancer among five cohort studies(RR = 1.04 95% CI [0.89, 1.22]). Both multivariable adjusted RR estimates with 95% CIs of each study and combined RR are shown in Figure 3.

### Meta-regression analysis and publication bias

**Figure 4:**
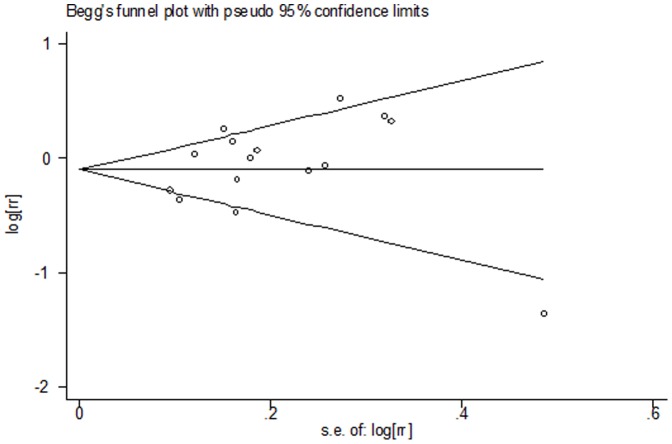
Funnel plot for publication bias in the studies investigating risk for ovarian cancer associated with fish intake.

To better investigate the possible sources of heterogeneity among case- control studies, a meta-regression analysis was performed. Study design, geographic area, control source, publication year, major confounders adjusted(total energy intake, use of oral contraceptives, parity, smoking status, alcohol consumption, family history of ovarian cancer), which may be potential sources of heterogeneity, were tested by a meta-regression method. We found that geographic area(0.021) and study design(P = 0.039) had statistical significance in a multivariate model. In the present meta-analysis, no publication bias was observed among studies using Begg's P value (P = 0.55); Egger's (P = 0.29) test, which suggested there was no evidence of publication bias (Figure 4).

## Discussion

The present meta-analysis included 15 observational studies currently available (five cohort studies and ten case–control studies), involving a total of 889,033 female subjects and 6,087 ovarian cancer cases. There was no significant heterogeneity among the five cohort studies, so a fixed-effect model was chosen, and we found that total fish consumption did not significantly affect the risk of ovarian cancer among cohort studies(comparing the highest with the lowest category). Because there was statistically significant heterogeneity among the 10 case-control studies, so a random-effect model was chosen. Finally, we found that total fish consumption did not significantly affect the risk of ovarian cancer among case-control studies. Meta-regression analysis revealed that geographic area and study design may be the source of heterogeneity. In our subgroup analyses, the results were not substantially affected by control source and most confounder adjustments(total energy intake, smoking status, alcohol consumption, and family history of ovarian cancer). We found that the association was substantially affected by geographic location where the studies conducted. Fish consumption was associated with a significant reduced risk of ovarian cancer among studies conducted in Europe and Australia. However, no significant association was detected among studies conducted in North America and Asia. There are many possible reasons which will lead to the difference in association between different areas. The differences in genetic susceptibility, culture, and lifestyles may explain part of the inconsistency of the results. Another reason is that the composition of total fish is different in different areas. Sensitivity analysis indicated that an omission of any studies did not alter the magnitude of observed effect significantly, suggesting a stability of our findings. Moreover, the results of Begg's test and Egger's test did not support the existence of major publication bias. In the present meta-analysis, we should notice that there were only five cohort studies investigating the association between fish intake and ovarian cancer risk. That number was rather low to draw firm conclusions. Compared with case-control studies, cohort studies are less susceptible to bias (e.g. recall bias, selection bias) due to their nature. Furthermore, case–control studies had a lower median quality score than cohort studies (5.5 versus 9). So more prospective cohort studies are needed to confirm this association in the future.

Fish consumption has both anticarcinoma and carcinogenic effects. On one hand, fish has protective effect against ovarian cancer. As we know, fish, especially fatty fish is rich in omega-3 fatty acids[37]. The results of Tavani A et al's meta-analysis showed that there was a significantly inverse association between intake of omega-3 polyunsaturated fatty acid and ovarian cancer risk[38]. Multiple mechanisms are involved in this chemopreventive activity, including cell growth inhibition and enhanced apoptosis, suppression of neoplastic transformation, and antiangiogenicity[39]–[41]. On the other hand, carcinogenic and mutagenic Nnitroso compounds and heterocyclic amines in processed fish may increase the risk of ovarian cancer[13], [14]. In the present meta-analysis, we only investigated total fish consumption and ovarian cancer risk. We thought that the combination of anticarcinoma effect from omega-3 fatty acids in fish and carcinogenic effect from carcinogenic and mutagenic Nnitroso compounds and heterocyclic amines in processed fish lead to the null association found in this meta-analysis. We haven't done subgroup analysis according to fish type(fresh fish and processed fish, fatty fish and nonfatty fish), for a lack of available studies. Among all the included studies, only the study conducted in Australia separated fatty and nonfatty fish. And they found that fatty fish intake was associated with a significant reduced risk of ovarian cancer (RR = 0.79, 95%CI [0.65, 0.98]), however, there was no significant association between nonfatty fish intake and ovarian cancer risk(RR = 0.86, 95%CI [0.75, 1.05]).

The strength of the present meta-analysis lies in a large sample size (889,033 female subjects and 6,087 ovarian cancer cases) and no significant evidence of publication bias. Two investigators independently performed the article identification, data extraction, and verification and resolved all discrepancies. Furthermore, our findings were stable and robust in sensitivity analyses. However, several limitations to this meta-analysis should be noted. Firstly, as a meta-analysis of observational data, the possibility of recall and selection biases cannot be ruled out. Compared with case-control studies, cohort studies are less susceptible to bias due to their nature. However, the present meta-analysis included only five cohort studies, so more prospective cohort studies are need to confirm the association in the future. Secondly, we did not search for unpublished studies, so only published studies were included in our meta-analysis. Therefore, publication bias may have occurred although no publication bias was indicated from both visualization of the funnel plot and Egger's test. Lastly, different types of fish(nonfatty fish and fatty fish, fresh fish and processed fish) may have different effects on the risk of ovarian cancer, however, we can't do detailed meta-analysis for a lack of available studies.

In conclusion, the present meta-analysis of five cohort and ten case-control studies showed that total fish consumption was not significantly associated with the risk of ovarian cancer. Further analysis on different fish species and food preparation methods should be conducted in future study.

## Supporting Information

Table S1 **Methodologic quality of studies included in the meta-analysis.**
(DOC)

Checklist S1 **PRISMA Checklist.**
(DOC)
