# Supplementary material for: Fish Intake and Ovarian Cancer Risk: A Meta-Analysis of 15 Case-Control and Cohort Studies
Source: PLoS One. 2014 Apr 14;9(4):e94601. doi: 10.1371/journal.pone.0094601 (PMC3986104; doi:10.1371/journal.pone.0094601)
Supplement: Table S1 — Methodologic quality of studies included in the meta-analysis. (DOC) [file pone.0094601.s001.doc]

**Table S1** Methodologic quality of studies included in the meta-analysis

**Table S1A** Methodologic quality of case-control studies included in the meta-analysis

| Authors | Publication year | Selection | | | | Comparability | | Exposure | | | Total quality scores |
| --- | --- | --- | --- | --- | --- | --- | --- | --- | --- | --- | --- |
|  |  | Adequate definition of cases | Representativeness of cases | Selection of control subjects | Definition of control subjects | Study controls for age / gender | Study controls for additional factors | Exposure assessment | Same method of ascertainment for cases and controls | Non-Response rate |  |
| Kolahdooz F | 2010 | ☆ | － | ☆ | － | ☆ | ☆ | ☆ | ☆ | ☆ | 7 |
| Hu J | 2008 | ☆ | ☆ | ☆ | － | ☆ | ☆ | ☆ | ☆ | ☆ | 8 |
| Pan SY | 2004 | ☆ | ☆ | ☆ | － | ☆ | ☆ | － | ☆ | ☆ | 7 |
| Yen ML | 2003 | ☆ | － | － | ☆ | ☆ | ☆ | － | － | ☆ | 5 |
| Zhang M | 2002 | ☆ | － | － | － | ☆ | ☆ | － | － | ☆ | 4 |
| Bosetti C | 2001 | ☆ | ☆ | ☆ | ☆ | ☆ | ☆ | ☆ | ☆ | － | 8 |
| Fernandez E | 1999 | ☆ | － | － | － | ☆ | ☆ | － | － | ☆ | 4 |
| Mori M | 1988 | ☆ | － | － | ☆ | ☆ | － | － | － | ☆ | 4 |
| La Vecchia C | 1987 | ☆ | － | － | － | ☆ | ☆ | － | － | ☆ | 4 |
| Cramer DW | 1984 | ☆ | － | ☆ | － | ☆ | － | ☆ | ☆ | ☆ | 6 |

**Table S1B** Methodologic quality of cohort studies included in the meta-analysis

| Authors | Publication year | Selection | | | | Comparability | | Outcome | | | Total quality scores |
| --- | --- | --- | --- | --- | --- | --- | --- | --- | --- | --- | --- |
|  |  | Representativeness of the exposed cohort | Selection of the unexposed cohort | Ascertainment of exposure | Demonstration that outcome of interest was not present at start of study | Study controls for age / gender | Study controls for additional factors | Assessment of outcome | Was follow-up long enough for outcomes to occur | Adequacy of follow up of cohorts |  |
| Gilsing AM | 2011 | ☆ | ☆ | ☆ | ☆ | ☆ | ☆ | ☆ | ☆ | ☆ | 9 |
| Daniel CR | 2011 | ☆ | ☆ | ☆ | ☆ | ☆ | ☆ | ☆ | ☆ | ☆ | 9 |
| Schulz M | 2007 | ☆ | ☆ | ☆ | ☆ | － | ☆ | ☆ | ☆ | ☆ | 8 |
| Kiani F | 2006 | ☆ | ☆ | － | ☆ | ☆ | ☆ | ☆ | ☆ | ☆ | 8 |
| Larsson SC | 2005 | ☆ | ☆ | ☆ | ☆ | ☆ | ☆ | ☆ | ☆ | ☆ | 9 |
